# Editorial: Neuromodulation of mood and eating behavior

**DOI:** 10.3389/fnins.2026.1798487

**Published:** 2026-03-04

**Authors:** Patricia de Gortari, Viridiana Alcántara-Alonso, Cinthia García-Luna

**Affiliations:** 1 Laboratorio de Neurofisiología Molecular, Instituto Nacional de Psiquiatria Ramon de la Fuente Muñiz, Mexico City, Mexico; 2 National Heart and Lung Institute, Imperial College London, London, United Kingdom

**Keywords:** depression, hedonic feeding, hepatokines, homeostatic feeding, mood disorders, polymorphisms, thyroid axis

For decades, psychiatry and endocrinology have been studied as independent systems; however, contemporary research is reconsidering these boundaries and revealing a sophisticated crosstalk between peripheral organs and the central nervous system. In this Frontiers Research Topic *Neuromodulation of mood and eating behavior*, eight groups of researchers explore and discuss how brain peptides, the activity of different brain regions, liver-derived signals, genomic polymorphisms and metabolic insults like high-fat diets (HFD) modulate neurobiological functions that influence mood and ingestive behaviors.

Cornejo and colleagues discuss in a mini-review the emergence of brain-acting hepatokines, such as IGF-1, FGF21, and GDF15, which shift the notion of the liver from a simple metabolic organ to a critical endocrine regulator of energy balance and brain function. The novelty of this liver-brain axis lies in the direct impact that these signals have on central pathways, providing a new perspective on how systemic metabolic disturbances can drive neurological outcomes (Giovanini et al.). In the same novelty line, de Gortari and colleagues showed that developmental timing dictates how metabolic challenges alter brain adaptation to them. The hypothalamic-pituitary-thyroid (HPT) axis, which regulates metabolic rate via thyrotropin-releasing hormone (TRH), shows a differential response to HFD-induced neuroinflammation in juvenile vs. adult animals. While both groups were exposed to a HFD, juvenile rats exhibit a greater impairment of HPT function and more rapid leptin resistance, partly due to an earlier and more pronounced elevation of the pro-inflammatory cytokine IL-1β in the brain. This suggests that the younger brain is uniquely vulnerable to long-term reprogramming by dietary fats, whereas adults show a relative resilience to cytokine overexpression despite similar metabolic stressors (Alvarez-Salas et al.).

Hong and collaborators evidenced the predictive power in the intersection of obesity and depression. In a significant study of Chinese adults, researchers developed a nomogram model that successfully identified high-risk individuals by integrating metabolic and physical markers like renal disease, grip strength, and sleep duration. With a depression prevalence of over 32% in the obese population studied, this predictive tool offers a moderate but vital discriminatory power for early clinical intervention (Yu et al.). Additionally, the diagnostic tool is further supported by the study performed by Proença da Fonseca and colleagues, in which they identify genomic deletions on the 16p11.2 region. In Brazilian cohorts, these polymorphisms, specifically those involving the SH2B1 gene, link severe early-onset obesity with metabolic syndrome and moderate binge-eating disorder, highlighting the clinical utility of genetic testing to distinguish monogenic forms of obesity from polygenic ones, and ensuring an appropriate therapy for those patients (da Silva Assis et al.).

The neurobiological substrates of mental and ingestive behaviors are localized in specific neuropeptidergic systems within the hypothalamus and the nucleus accumbens, among other regions. For instance, Fu and colleagues discuss about the potential therapeutic use of the tuberoinfundibular peptide of 39 residues (TIP39), which acts through the PTH2R receptor, and that has emerged as a pluripotent molecule essential for mood regulation and maternal behavior. Its deficiency is now considered a potential pathway for the pathophysiology of postpartum depression (PPD), offering a novel therapeutic entry point for treating maternal care deficits (Wang, Liu et al.). Similarly, Jiang and colleagues discuss the role of hypocretin (orexin) in metabolic and mood disorders and its therapeutic potential. Hypocretin system serves as a master physiological integrator, linking arousal and emotional states to energy metabolism. While its role in wakefulness is well-known, its influence on feeding is paradoxically demonstrated in narcolepsy patients who gain weight despite lower caloric intake, illustrating the necessity to further study hypocretin and unravel its role in maintaining metabolic homeostasis. Moreover, hypocretin has been allocated with a role in stress resilience and anxiety and depression disorders; thus, it may also have a therapeutic utility in the treatment of those pathologies (Wang, Fu et al.). Regarding the hedonic aspects of feeding, the nucleus accumbens serves as a critical integration hub where the homeostatic signals interact with hedonic drive. Calafiore and collaborators show that injections of neuropeptide Y (NPY) and orexin-A into this region significantly increase the intake of palatable, sweetened fat diets, particularly when they act synergistically with μ-opioid receptor stimulation. This reinforces the idea that the brain's reward centers do not operate independently to other signals, and in turn they are constantly regulated by homeostatic inputs (Pratt et al.). Li and colleagues show that even in aquatic models like tilapia, the relationship between fatty acids and brain signaling remains conserved; short-term fasting reduces the anorexigenic POMC signal, while eicosapentaenoic acid (EPA) compensation can suppress food intake and inhibit AMPK pathways to restore homeostasis (Yu et al.).

Collectively, these findings suggest that whether through the liver, the thyroid axis, neuropeptidergic systems, or specific genomic markers, metabolic health is intimately linked to mental wellbeing, emphasizing the necessity of a more integrated approach to metabolic-psychiatric research.

